# Neuroendoscopic hematoma evacuation vs. craniotomy in hypertensive intracerebral hemorrhage: a retrospective comparative study on surgical efficiency and long-term functional outcomes

**DOI:** 10.3389/fsurg.2025.1670479

**Published:** 2025-10-27

**Authors:** Xinyun Ye, Guanlin Huang, Jing Hu, Wentao Lai

**Affiliations:** 1 Department of Neurosurgery, Ganzhou People's Hospital, Ganzhou, China; 2 Department of Hematology, Ganzhou People's Hospital, Ganzhou, China

**Keywords:** hypertensive intracerebral hemorrhage, neuroendoscopic surgery, open craniotomy, operative efficiency, functional recovery, quality of life

## Abstract

**Objective:**

To investigate the impact of neuroendoscopic surgery on surgical efficiency and long-term functional outcomes in patients with hypertensive intracerebral hemorrhage (HICH).

**Methods:**

This retrospective comparative study was conducted on a cohort of 60 patients diagnosed with HICH who were admitted to Ganzhou People's Hospital between January 2020 and December 2022. The patients were divided into two groups based on the surgical technique employed: neuroendoscopic hematoma evacuation (NEHE, *n* = 30) and traditional craniotomy hematoma evacuation (CHE, *n* = 30). Primary outcomes measured included operative time, intraoperative blood loss, hematoma clearance rate, and long-term functional recovery assessed at the one-year follow-up using the Stroke-Specific Quality of Life Scale (SS-QOL), Modified Barthel Index (MBI), and Fugl-Meyer Assessment (FMA).

**Results:**

The NEHE group demonstrated statistically significant improvements in surgical efficiency and safety. Specifically, the operative time was reduced by 25% (93.75 ± 10.56 min vs. 124.66 ± 21.71 min, *p* < 0.001), and intraoperative blood loss decreased by 44% (30.32 ± 5.63 mL vs. 53.75 ± 10.56 mL, *p* < 0.001), indicating markedly lower surgical trauma compared to CHE. Notably, the hematoma clearance rate in the NEHE group (84.66 ± 7.33%) surpassed that of CHE (80.21 ± 8.54%, *p* = 0.03), which may correlate with enhanced visualization of residual clots under endoscopic guidance. At 1-year follow-up, NEHE patients exhibited superior functional recovery, with SS-QOL scores increasing by 13% (156.74 ± 26.64 vs. 138.22 ± 34.45, *p* = 0.03), MBI scores by 20% (59.34 ± 11.51 vs. 49.22 ± 16.71, *p* = 0.01), and FMA scores by 23% (35.27 ± 3.98 vs. 28.63 ± 5.72, *p* < 0.001). Crucially, stratified analysis revealed maximal functional benefits in basal ganglia hemorrhages where FMA scores were 27% higher with NEHE (37.12 ± 3.15 vs. 29.23 ± 4.82, *p* < 0.001), contrasting with non-significant differences in lobar hemorrhages (*p* = 0.41).

**Conclusion:**

In summary, our findings affirm that NEHE provides superior surgical outcomes and a favorable safety profile in the management of HICH, with significant improvements noted in long-term quality of life and motor function. The results advocate for the adoption of NEHE as a primary approach for HICH cases.

## 1 Introduction

Hypertensive intracerebral hemorrhage (HICH) remains a devastating neurological emergency with a 30-day mortality rate exceeding 40% (1). Characterized by spontaneous rupture of small penetrating arteries in the brain parenchyma, HICH induces hematoma formation and mass effect, leading to irreversible neurological deficits if not promptly managed (1, 2). Despite advancements in medical therapy, surgical intervention remains a cornerstone for patients with significant hematoma volume (≥30 mL) or deteriorating consciousness (1, 3). Traditional craniotomy, while effective in hematoma evacuation, is associated with prolonged operative time, substantial blood loss, and iatrogenic brain injury due to extensive tissue retraction (4). In contrast, minimally invasive techniques, particularly neuroendoscopic hematoma evacuation (NEHE), have gained traction for their potential to reduce surgical trauma (1, 4). A growing number of studies have confirmed that NEHE achieves comparable hematoma clearance to craniotomy while shortening hospitalization duration (5–8). However, existing studies predominantly focus on short-term outcomes (e.g., 30-day mortality, postoperative complications), leaving critical gaps in understanding long-term functional recovery and quality of life (QoL).

Despite growing evidence supporting minimally invasive techniques in HICH management, comprehensive evaluations of their multidimensional outcomes remain scarce. The primary objective of this retrospective study was to investigate the surgical efficiency (operative time, intraoperative blood loss, and hematoma clearance rate) and safety profile (rebleeding rates) of neuroendoscopic surgery in patients with HICH, while also evaluating its long-term prognostic impact through 1-year follow-up assessments across three critical domains: health-related quality of life (Stroke-Specific Quality of Life Scale, SS-QOL), independence in activities of daily living (Modified Barthel Index, MBI), and motor functional recovery (Fugl-Meyer Assessment, FMA).

## 2 Materials and methods

### 2.1 Study design and ethical considerations

**Figure 1:**
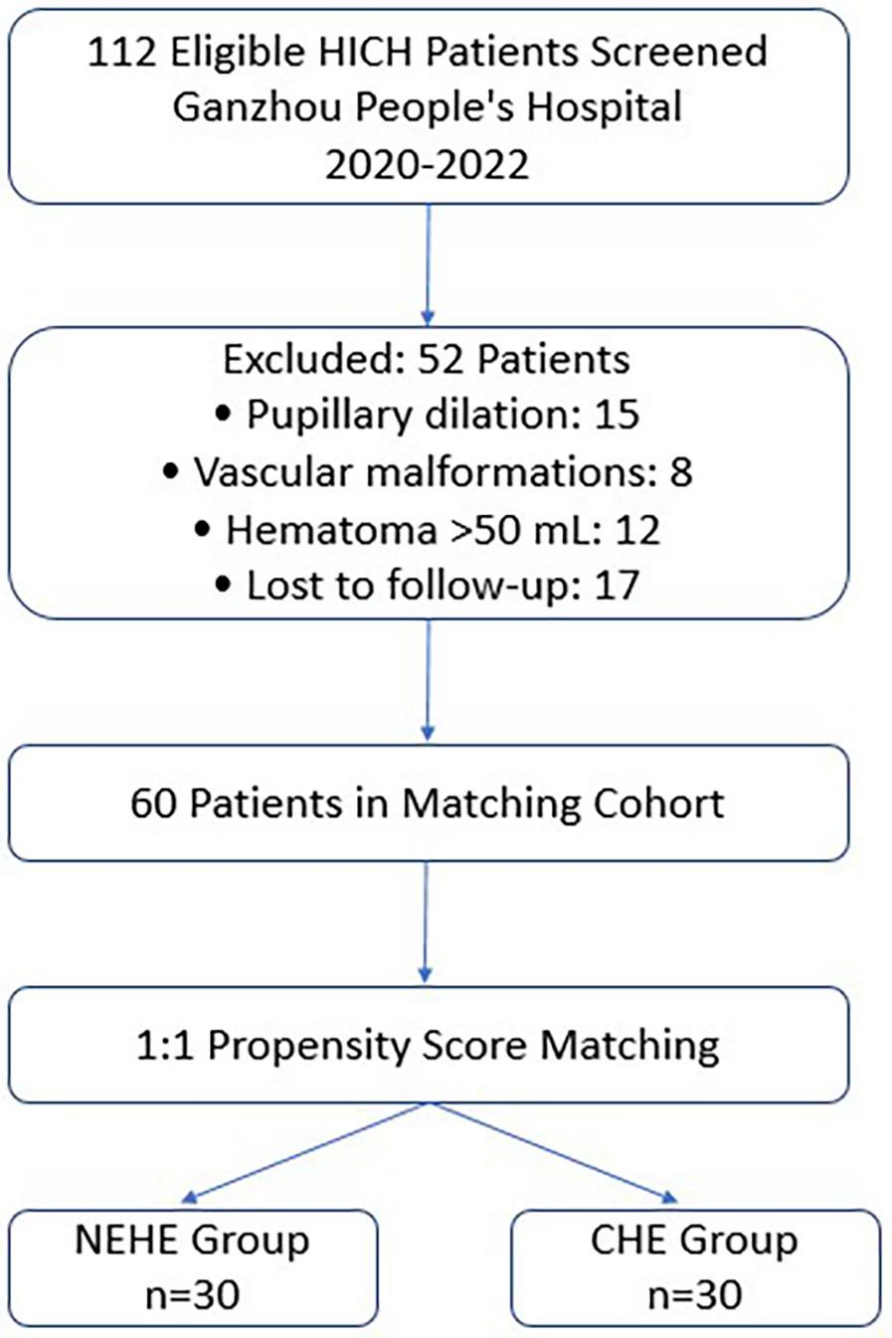
Flowchart of patient selection and propensity score matching process. HICH, hypertensive intracerebral hemorrhage; NEHE, neuroendoscopic hematoma evacuation; CHE, craniotomy hematoma evacuation.

This single-center retrospective comparative study enrolled 112 patients with HICH admitted to the Department of Neurosurgery at Ganzhou People's Hospital between January 2020 and December 2022. From an initial pool of 112 eligible HICH patients, we performed 1:1 propensity score matching (PSM) using logistic regression with covariates including age, hematoma volume, GCS score, and hemorrhage location. Caliper width was set at 0.2 SD of the propensity score logit. This yielded 30 matched pairs (NEHE vs. CHE) with standardized differences <10% for all baseline variables (Figure 1). Participants were divided into NEHE (*n* = 30) and CHE (*n* = 30) groups. The study protocol was approved by the Institutional Ethics Committee of Ganzhou People's Hospital (Approval No. GZPH-2023-NS-045), and written informed consent was waived due to the retrospective design.

Inclusion criteria: Diagnosis of supratentorial HICH confirmed by cranial CT, with hematoma volume 30–50 mL; Normal coagulation profile (INR ≤1.2, platelet count ≥100 × 10^9^/L); Availability of complete 1-year follow-up data.

Exclusion criteria: Pupillary dilation indicating brain herniation; Secondary hemorrhage from vascular malformations or aneurysms; Severe comorbidities (e.g., renal failure, advanced cancer); Cognitive impairment unrelated to HICH.

Surgical Indications: Surgical intervention was indicated for patients with HICH presenting with a hematoma volume between 30 and 50 mL, accompanied by a midline shift of ≥5 mm and/or clinical deterioration (a decrease in Glasgow Coma Scale score by ≥2 points). Patients with smaller hematomas without significant mass effect or neurological decline were managed conservatively and were not included in this surgical cohort.

### 2.2 Surgical procedures

#### 2.2.1 Neuroendoscopic hematoma evacuation (NEHE group)

**Figure 2:**
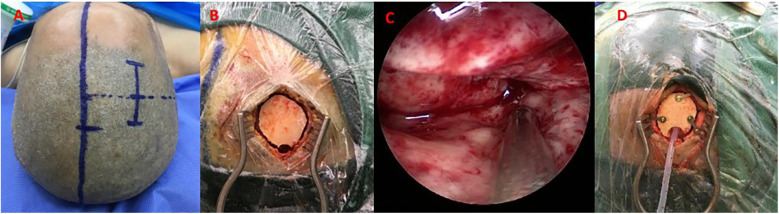
Neuroendoscopic hematoma evacuation assisted by small bone window. **(A)** A straight incision approximately 5 cm in length; **(B)** A small bone window (approximately 2 × 3 cm); **(C)** Neuroendoscopic evacuation using a 4-mm rigid endoscope (Karl Storz); **(D)** Placement of a drainage tube in the operative area.

**Figure 3:**
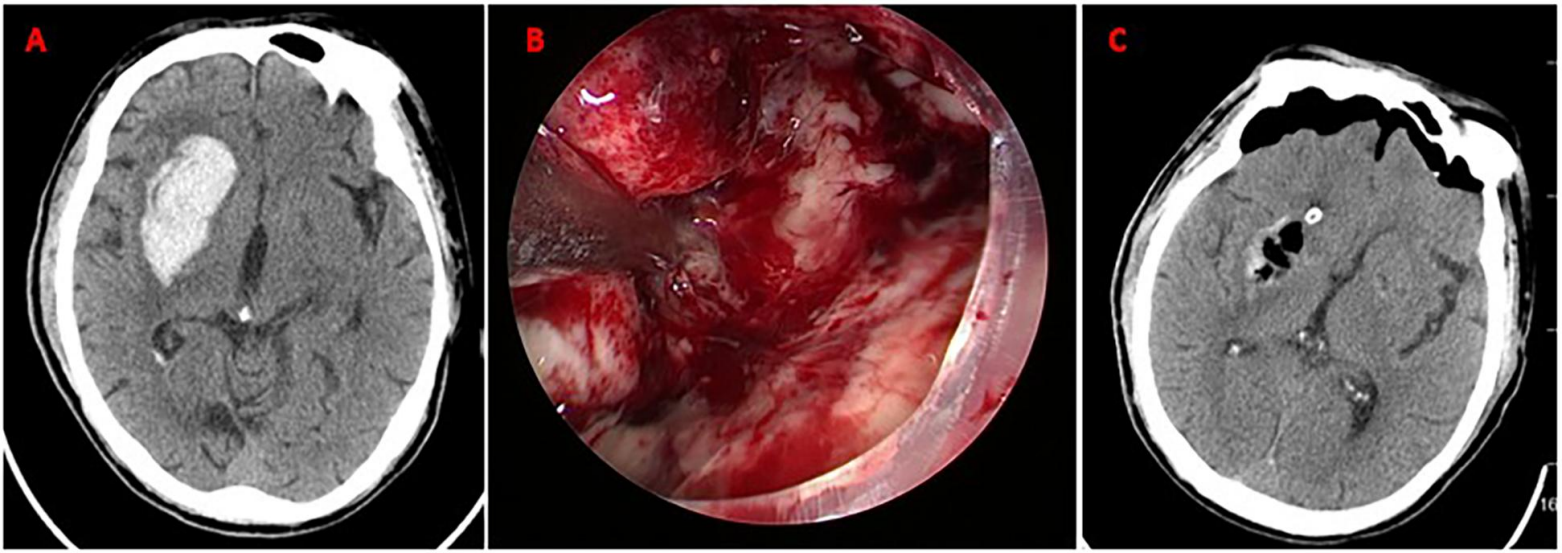
Neuroendoscopic evacuation of right basal ganglia hematoma. **(A)** Axial CT demonstrates acute hypertensive hemorrhage (volume: 35 mL) in the right basal ganglia; **(B)** Intraoperative neuroendoscopic view during hematoma evacuation via a frontal approach. The hematoma cavity is visualized; **(C)** Postoperative axial CT scan confirming near-total hematoma evacuation (residual volume <2 mL). No new ischemic lesions or rebleeding are observed. Mild perihematomal edema is present.

Under general anesthesia, the surgical approach (temporal or frontal) was determined based on preoperative CT localization of hematoma morphology and proximity to critical structures (e.g., avoiding lateral fissure vessels). A 4 cm linear skin incision was made, followed by a burr hole (diameter: 1.5–3.0 cm) to create a mini-craniotomy. After dural incision, a 4-mm rigid neuroendoscope (Karl Storz, Germany) with 0° angled lens was used for hematoma visualization. Hematoma evacuation was performed using suction and irrigation. Following hematoma evacuation, continuous irrigation with isotonic saline (37°C) was administered to maintain optimal visualization and minimize thermal injury potential. Active arterial bleeding was controlled using bipolar electrocautery, while venous oozing was managed with absorbable fluid gelatin. A subdural drain was placed before layered closure. The specific operation process is shown in Figure 2 and the perioperative images of typical cases are shown in Figure 3.

#### 2.2.2 Craniotomy hematoma evacuation (CHE group)

**Figure 4:**
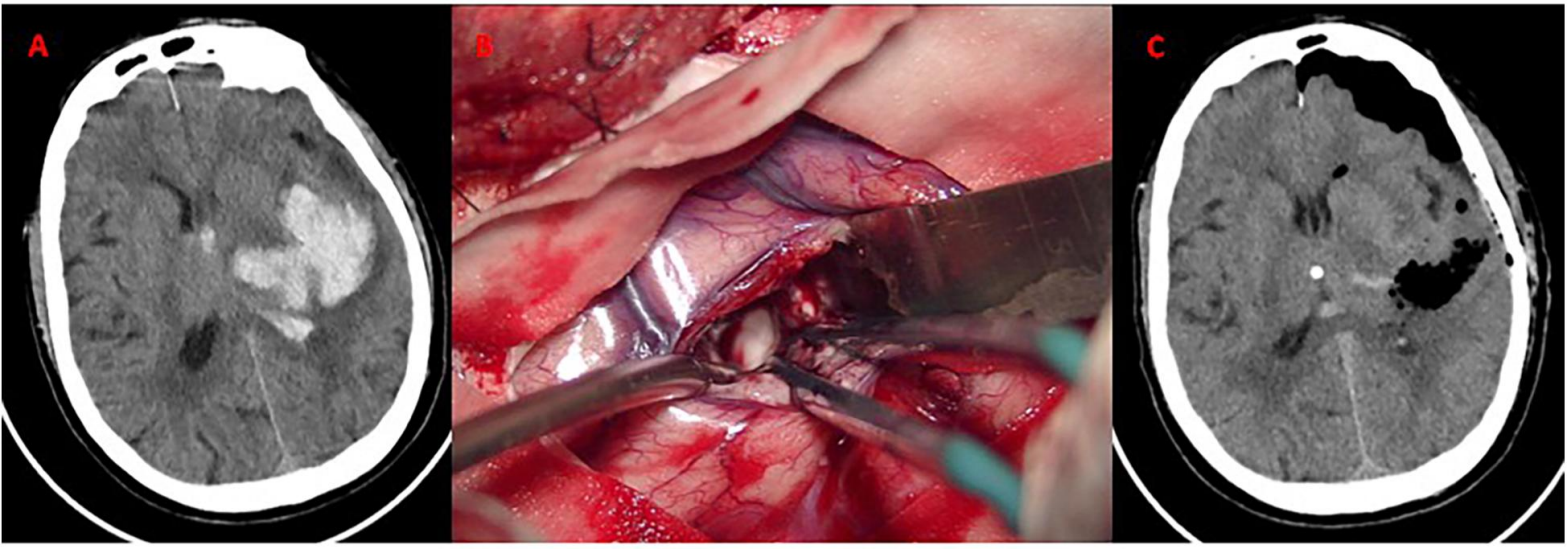
Craniotomy evacuation of left basal ganglia hematoma. **(A)** Axial CT demonstrates acute hypertensive hemorrhage (volume: 30 mL) in the left basal ganglia; **(B)** Intraoperative microscopic view. Hematoma evacuation via a standard trauma craniotomy (6 × 8 cm bone flap). The bone flap was successfully repositioned without decompressive craniectomy. **(C)** CT confirms near-total hematoma evacuation. No new ischemic lesions or rebleeding observed.

A standard trauma flap was designed based on hematoma location. Following a larger craniotomy (6–8 cm diameter), hematoma evacuation was performed under microscopic guidance. Hemostasis was achieved using bipolar cautery and absorbable hemostatic agents. Suture the dural membrane and replace the bone flap based on intraoperative findings and cerebral edema status. The perioperative images of typical cases are shown in Figure 4.

### 2.3 Postoperative rehabilitation

Both groups initiated standardized rehabilitation protocols on postoperative day 7 (Day 0 = surgery day), including: Limb positioning and passive mobilization: 30 min/session, twice daily; Muscle strength and coordination training: Resistance exercises using elastic bands, 45 min/day; Gait and balance training: Overground walking with parallel bars, 20 min/day; Neuromuscular electrical stimulation parameters (frequency: 50 Hz; pulse width: 250 μs) were adjusted based on spasticity assessed via Modified Ashworth Scale. The 4-week program was supervised by licensed physiotherapists, with adherence monitored via attendance logs.

### 2.4 Outcome measures and statistical analysis

Preoperative and postoperative hematoma volumes were calculated using the ABC/2 method on non-contrast CT scans. All measurements were performed independently by two experienced neurosurgeons, and any discrepancies were resolved by consensus. Surgical data, including operative time (minutes), intraoperative blood loss (mL), hematoma clearance rate [calculated as (preoperative volume −postoperative residual)/preoperative volume × 100%], were collected from all patients. Postoperative cranial CT scans were performed to confirm the presence or absence of rebleeding (defined as symptomatic hemorrhage confirmed by CT within 72 h).

At the 1-year follow-up, the SS-QOL, MBI, and FMA score were utilized for assessment. Higher scores indicate better quality of life, daily living abilities, and limb motor function.

### 2.5 Statistical methods

Continuous variables were tested for normality using Shapiro–Wilk tests. Normally distributed data (age, hematoma volume) were compared via independent t-tests, reported as mean ± SD; non-normal data (operative time) were analyzed using Mann–Whitney U tests, presented as median (IQR). Categorical variables (sex, hemorrhage location) were compared with *χ*^2^ or Fisher's exact tests. Bonferroni correction was applied for multiple comparisons involving three functional outcomes (SS-QOL, MBI, FMA). Subgroup analyses employed Welch's *t*-test for basal ganglia hemorrhages (heterogeneous variances) and independent t-test for lobar hemorrhages (homogeneous variances per Levene's test). Bonferroni correction adjusted for two subgroup comparisons (*α* = 0.025). Analyses were performed using SPSS 26 (IBM Corp., USA), with *p* < 0.05 considered statistically significant.

## 3 Results

### 3.1 Baseline demographic and clinical characteristics

A total of 60 patients with hypertensive intracerebral hemorrhage were enrolled according to inclusion and exclusion criteria, with 30 cases assigned to the NEHE group and 30 cases to the CHE group (Table 1). No significant differences were observed between the two groups in baseline characteristics, including age (NEHE group: 63.41 ± 4.22 years vs. CHE group: 65.2 ± 5.01 years, *p* = 0.14), sex distribution (male/female ratio: 18/12 vs. 16/14, *p* = 0.60), hemorrhage location (basal ganglia: 46.7% vs. 40.0%; frontal lobe: 20.0% vs. 16.7%; occipital lobe: 23.3% vs. 26.7%; cerebellum: 10.0% vs. 16.7%, *p* = 0.94), preoperative hematoma volume (42.85 ± 6.24 mL vs. 45.37 ± 5.68 mL, *p* = 0.11), intraventricular extension (26.7% vs. 23.3%, *p* = 0.77), or preoperative Glasgow Coma Scale (GCS) scores (10.4 ± 3.1 vs. 10.2 ± 3.9, *p* = 0.83). All baseline parameters were statistically comparable (*p* > 0.05).

**Table 1:** Baseline characteristics of patients underwent hematoma evacuation.

Variables	NEHE group *n* = 30	CHE group *n* = 30	*t*/*χ*^2^	*P*
Age (yrs)	63.41 ± 4.22	65.2 ± 5.01	1.50	0.14
Gender			0.27	0.60
Male (*n*, %)	18	16		
Female (*n*, %)	12	14		
Position of hemorrhage			0.81	0.94
Basal ganglia	14	12		
Frontal lobe	6	5		
Occipital lobe	7	8		
Cerebellum	3	5		
Hematoma breaking into the ventricle (*n*)	8	7	0.09	0.77
Hematoma volume (mL)	42.85 ± 6.24	45.37 ± 5.68	1.64	0.11
GCSscore before surgery	10.4 ± 3.1	10.2 ± 3.9	0.22	0.83

### 3.2 Comparative analysis of surgical outcomes: neuroendoscopic vs. craniotomy

Surgical efficacy parameters are summarized in Table 2. the operative time was reduced by 25% (93.75 ± 10.56 min vs. 124.66 ± 21.71 min, *p* < 0.001), and intraoperative blood loss decreased by 44% (30.32 ± 5.63 mL vs. 53.75 ± 10.56 mL, *p* < 0.001), indicating markedly lower surgical trauma compared to CHE. Moreover, the hematoma evacuation rate was higher in the neuroendoscopic group (84.66 ± 7.33% vs. 80.21 ± 8.54%, *t* = 1.82, *p* = 0.03). Although re-bleeding occurred in 2 cases (6.7%) in the NEHE group and 3 cases (10.0%) in the CHE group, no significant difference was observed in postoperative re-hemorrhage rates (*p* = 0.69). In the NEHE group, two patients died due to pulmonary infections. In the CHE group, four deaths occurred, including two cases attributed to pulmonary infections and two cases caused by postoperative secondary cerebral edema.

**Table 2:** Comparison of surgical efficacy.

Group	Operative time (min)	Intraoperative blood loss (mL)	Hematoma evacuation rate (%)	Re-bleeding (*n*, %)
NEHE Group	93.75 ± 10.56	30.32 ± 5.63	84.66 ± 7.33	2 (6.7%)
CHE group	124.66 ± 21.71	53.75 ± 10.56	80.21 ± 8.54	3 (10.0%)
*t*/*χ*^2^	7.01	10.72	1.82	0.00
*P*	<0.001	<0.001	0.03	1*

* Intergroup differences in rebleeding were analyzed using Fisher's exact test (two-sided).

**Table 3:** Comparison of SS-QOL, MBI, and FMA.

Group	SS-QOL	MBI	FMA
NEHE group *N* = 28	156.74 ± 26.64	59.34 ± 11.51	35.27 ± 3.98
CHE group *N* = 26	138.22 ± 34.45	49.22 ± 16.71	28.63 ± 5.72
*t*	2.21	2.61	4.98
*P*	0.03	0.01	<0.001

### 3.3 Long-term functional recovery and quality of life assessments

All patients underwent standardized postoperative rehabilitation. At 1-year follow-up, NEHE patients exhibited superior functional recovery, with SS-QOL scores increasing by 13% (156.74 ± 26.64 vs. 138.22 ± 34.45, *p* = 0.03), MBI scores by 20% (59.34 ± 11.51 vs. 49.22 ± 16.71, *p* = 0.01), and FMA scores by 23% (35.27 ± 3.98 vs. 28.63 ± 5.72, *p* < 0.001), reflecting clinically meaningful improvements in daily living independence and motor function. The detailed comparative results are summarized in Table 3.

### 3.4 Stratified analysis by hemorrhage location

Stratified analysis by hemorrhage location revealed differential treatment effects (Table 4). For basal ganglia hemorrhages—representing nearly half our cohort (46.7%)—NEHE demonstrated pronounced FMA superiority (37.12 ± 3.15 vs. 29.23 ± 4.82, *p* < 0.001), equivalent to a 27% functional gain. Conversely, lobar hemorrhages showed no significant intergroup difference (34.50 ± 4.32 vs. 32.85 ± 5.71, *p* = 0.41), suggesting anatomical location mediates NEHE's benefit magnitude.

**Table 4:** Stratified analysis of Fugl-Meyer assessment (FMA) scores by hemorrhage location.

Hemorrhage location	Group	*n*	FMA score (mean ± SD)	*t*	*P*
Basal ganglia	NEHE	14	37.12 ± 3.15	4.85	<0.001
	CHE	12	29.23 ± 4.82		
Lobar^a^	NEHE	13	34.50 ± 4.32	0.83	0.41
	CHE	13	32.85 ± 5.71		

NEHE, neuroendoscopic hematoma evacuation; CHE, craniotomy hematoma evacuation.

a Lobar hemorrhages include frontal (NEHE = 6, CHE = 5) and occipital (NEHE = 7, CHE = 8) locations.

## 4 Discussion

This retrospective comparative study demonstrates that NEHE significantly surpasses traditional craniotomy (CHE) in both surgical efficiency and long-term functional recovery for patients with supratentorial hypertensive intracerebral hemorrhage (HICH). Our findings reveal markedly reduced operative time and blood loss, improved hematoma clearance, critically, superior gains in health-related quality of life, activities of daily living independence, and motor function sustained at 1-year follow-up. Against this backdrop of significant morbidity and unmet needs in long-term functional recovery, our findings demonstrate that NEHE offers a promising approach to improve the trajectory for HICH patients. Specifically, comparative analysis revealed that NEHE significantly outperformed traditional craniotomy (CHE) in both surgical efficiency and sustained functional outcomes. The 23% higher FMA score in the NEHE group translates to clinically meaningful motor recovery. According to Fugl-Meyer criteria (9), scores >34 indicate moderate limb functionality (e.g., voluntary grasp and release), whereas CHE scores (mean 28.6) fall within the severe impairment range. Similarly, the 20% improvement in MBI exceeds the MCID of 10 points, SS-QOL gain (18.52 points) represents a 13% quality-of-life enhancement, signifying a transition from moderate dependence to mild dependence in daily activities (10).

Superior functional outcomes are mechanistically linked to two synergistic advantages of NEHE: minimized white matter injury and enhanced hematoma evacuation completeness. It likely stems from fundamental differences in surgical approach that minimize iatrogenic injury to critical white matter tracts. Neuroendoscopic techniques enable a parallel surgical trajectory along the longitudinal axis of white matter fibers, thereby minimizing axonal disruption and subsequent secondary brain injury through targeted, anatomy-respecting dissection planes (11, 12). This trajectory exploits natural dissection planes during hematoma evacuation, with diffusion tensor imaging tractography confirming smaller angular deviation from principal fiber orientations. In stark contrast, traditional craniotomy necessitates a transgyral approach that traverses the corona radiata at substantial angles to CST fibers. This oblique trajectory generates significantly greater axonal shear strain, quantified by a higher reduction in fractional anisotropy, reflecting pronounced microstructural damage to white matter pathways (13). It likely stems from the compact fiber organization in the posterior limb creates “surgical vulnerability zones” where traditional retraction causes irreversible damage. Beyond minimizing structural damage to white matter pathways, the technical advantages of neuroendoscopy also contributed to a more complete evacuation of the hematoma itself, a critical factor influencing outcomes. Hematoma volume was identified as an independent predictor of 30-day mortality in the widely validated HICH grading system, underscoring its critical role in prognostication for intracerebral hemorrhage outcomes (14). Consequently, hematoma evacuation rate is a critical determinant of survival and prognosis in cerebral hemorrhage patients, serving as a crucial indicator for evaluating surgical efficacy (15, 16). Our findings demonstrate that the higher hematoma clearance rate in the NEHE group contrasts with earlier studies showing equivalence between techniques, indicating superior completeness of hematoma removal with neuroendoscopic surgery. Enhanced illumination and broader surgical field under endoscopy, enabling precise visualization of deep hematoma margins, minimizing blind dissection compared to craniotomy, which makes the treatment more thorough and at the same time has the advantage of less damage (14). Conventional craniotomy may result in imprecise hematoma evacuation and hemostatic control, potentially exacerbating iatrogenic injury to white matter tracts through indiscriminate tissue retraction (17). The more complete hematoma evacuation achieved with NEHE reduces mass effect and limits exposure to neurotoxic blood degradation products (e.g., thrombin, free hemoglobin), which are key mediators of secondary brain injury and perihematomal edema (2, 18, 19). While we did not directly measure edema volume, this mechanistic advantage, combined with the reduced axonal shear strain from endoscopic parallel trajectories, synergistically contributes to preserving peri-lesional neural tissue integrity. Critically, these dual mechanisms operate synergistically to enhance functional recovery. This mechanistic synergy - neuroanatomical preservation coupled with pathological burden reduction - collectively establishes the neurophysiological foundation for superior long-term functional outcomes.

Our stratified analysis provides critical insights into the differential treatment effects observed across hemorrhage locations (Table 4). We found that basal ganglia hematomas, comprising 46.7% of our cohort, derive maximal benefit from endoscopic techniques. The pronounced 27% FMA advantage of NEHE in basal ganglia hemorrhages validates the white matter protection hypothesis proposed earlier. The compact fiber architecture of the posterior limb creates surgical vulnerability zones where traditional trans-fissure approaches cause irreversible CST damage. Conversely, the non-significant difference in lobar hemorrhages suggests cortical reorganization may compensate for surgical trauma in these regions. This aligns with diffusion tensor imaging studies showing greater neuroplastic potential in cortical vs. subcortical pathways after injury (20). The anatomical specificity of NEHE's benefit underscores its particular value for deep hemorrhages - precisely those with historically worst functional outcomes. These findings corroborate multicenter registry data showing 38% greater mobility recovery in basal ganglia hemorrhages treated endoscopically (21), while explaining the equivocal lobar hemorrhage results in trials like MISTIE III (22). The location-dependent efficacy emphasizes that surgical innovation must account for neuroanatomical context, not merely hematoma volume.

Complementing the benefits of enhanced evacuation completeness and reduced neural injury, NEHE also demonstrated significant gains in surgical efficiency, which contribute to reduced perioperative stress and potentially faster initiation of rehabilitation. NEHE demonstrated significant improvements in surgical efficiency compared to conventional craniotomy, achieving a 25% reduction in operative time and 44% decrease in intraoperative blood loss. Reduced operative time not only lowers anesthesia exposure but enables earlier ICU mobilization, potentially reducing pneumonia risk (23). And the shorter operative time facilitates earlier rehabilitation initiation. The minimal cortical exposure may explain the 44% lower blood loss. These gains primarily stem from three synergistic innovations: First, the minimally invasive keyhole approach (1.5–3 cm bone window) reduced cortical exposure and eliminated time-consuming extensive bone window craniotomy and dural tack-up sutures. Second, 4 K endoscopic visualization with 30° scope rotation capability enabled real-time identification of deep hematoma margins and enhancing hematoma clearance to 84.66%. Third, employing the chopstick technique under endoscopic visualization enhanced hemostatic efficiency in deep-seated hemorrhagic sites, reducing mean hemostasis time.

Importantly, these significant advantages in efficacy and efficiency were achieved without compromising procedural safety. Despite higher evacuation rates, rebleeding remained comparable, attributable to precise bipolar coagulation under endoscopic guidance (20). Mortality equivalence aligns with MISTIE III findings (22), though sample size limits subgroup analysis for massive hemorrhages (>50 mL). This aligns with existing evidence that minimally invasive neuroendoscopy achieves hematoma evacuation efficacy comparable to craniotomy while maintaining equivalent safety in hemorrhage control (21). This phenomenon can be primarily ascribed to the enhanced hemostatic efficacy achieved through bipolar coagulation under real-time neuroendoscopicc visualization, which enables precise anatomical targeting while minimizing collateral thermal injury to perilesional neural structures. The mortality events primarily resulted from postoperative cerebral edema and pulmonary infections. In the NEHE group, both deaths resulted from pulmonary infections, a common complication in critically ill neurosurgical patients often linked to prolonged immobilization or ventilator dependence (24). In contrast, the CHE group experienced four deaths: two similarly due to pulmonary infections, and two attributed to postoperative secondary cerebral edema. The occurrence of fatal cerebral edema exclusively in the craniotomy cohort warrants consideration. While cerebral edema is a known sequela of intracerebral hemorrhage itself, its severity can be exacerbated by surgical trauma. The more extensive tissue manipulation, longer operative times, and potentially greater retraction injury inherent to open craniotomy may contribute to increased perihematomal edema and intracranial pressure postoperatively (2, 25). Although preoperative hematoma volumes were comparable, the trend toward larger volumes in the CHE group could have interacted with surgical approach to elevate edema risk. Consequently, the two edema-related deaths in the CHE group may reflect, at least in part, the consequences of greater surgical invasiveness. These observations, while derived from a small number of events, underscore that the reduced surgical trauma associated with NEHE, evidenced by shorter operative times and minimal cortical exposure, may translate not only to improved functional outcomes but also to a lower risk profile for severe, life-threatening complications like refractory cerebral edema, particularly in vulnerable patients.

While randomized trials have established the procedural safety of neuroendoscopic evacuation in the acute phase (22, 26), evidence regarding its long-term functional benefits remained limited prior to this investigation. Our study addresses this critical knowledge gap by demonstrating sustained functional superiority at 1-year follow-up - a clinically relevant timeframe for neurorehabilitation. We attribute this enhanced recovery profile to the white matter preservation capabilities of neuroendoscopic techniques, which create a more permissive environment for neuroplasticity during rehabilitation. This functional advantage carries significant implications for clinical practice guidelines. Current AHA/ASA recommendations prioritize mortality reduction in HICH management (27), yet our findings reveal that neuroendoscopic surgery uniquely addresses the equally vital need for functional independence, evidenced by substantially more patients achieving clinically meaningful independence thresholds. This represents a paradigm shift from survival-focused to function-oriented surgical decision-making. Therefore, NEHE represents a superior surgical strategy for HICH, offering significant advantages in procedural efficiency and, crucially, leading to substantially improved long-term functional independence and quality of life compared to conventional craniotomy.

These functional advantages, however, must be contextualized within the technical constraints of both approaches and individualized patient factors. NEHE's efficacy is contingent upon operator expertise, requiring adequate cases to overcome the steep learning curve associated with limited instrument maneuverability in complex hematoma architectures. Hemostatic control remains challenging in coagulopathic patients (INR > 1.5), where restricted bipolar access angles correlate with rebleeding risk. Based on our findings, we propose an individualized risk-benefit framework for surgical selection. NEHE is preferentially indicated for supratentorial hemorrhages (30–60 mL) with preserved consciousness (GCS ≥ 8), particularly deep-seated basal ganglia/thalamic lesions. Optimal outcomes require confirmed anticoagulation reversal (INR ≤ 1.3) and avoidance in posterior fossa pathologies. Craniotomy remains essential for lobar hemorrhages with >50% cortical involvement, cerebellar hematomas >15 mL causing brainstem compression, and scenarios requiring ongoing hemostatic support. It also serves as salvage therapy for endoscopic failures requiring intraoperative conversion. Notwithstanding these considerations, NEHE represents a transformative approach for eligible candidates, achieving the dual objectives of procedural efficiency and optimized functional recovery.

While our findings demonstrate significant advantages of neuroendoscopic evacuation, several methodological constraints merit consideration. The single-center design and moderate sample size (*n* = 60) may limit generalizability, particularly for less common hemorrhage locations (e.g., cerebellar cases comprising 10%–16.7% of our cohort). The retrospective nature introduces potential selection bias despite propensity score matching. Furthermore, the 1-year follow-up period, while capturing key functional milestones, may be insufficient to evaluate long-term cognitive trajectories. To address these limitations, future multicenter randomized trials should: (1) incorporate diffusion tensor imaging to quantitatively assess axonal integrity preservation as a predictor of motor recovery; (2) extend follow-up to 3–5 years with comprehensive neuropsychological batteries to evaluate domain-specific cognitive outcomes; And (3) incorporate serial edema volumetry to elucidate its contribution to functional outcomes. Such methodological refinements would validate our observed functional advantages while elucidating the neuroanatomical substrates underlying recovery.

## 5 Conclusion

NEHE demonstrates superior surgical efficacy over craniotomy, achieving significant reductions in operative time and intraoperative blood loss while improving hematoma clearance rates. Critically, NEHE translates into sustained functional recovery, with superior outcomes in SS-QOL, activities of daily living, and motor function. Superior hematoma clearance, coupled with minimized white matter injury, collectively underpins the enhanced functional recovery observed after NEHE. Furthermore, while overall mortality and rebleeding rates were comparable between groups, the occurrence of fatal postoperative cerebral edema exclusively in the craniotomy cohort suggests a potential safety advantage for NEHE in mitigating severe edema-related complications associated with greater surgical trauma. Therefore, NEHE is recommended as the preferred approach for supratentorial HICH, particularly in basal ganglia hemorrhages, where the most significant functional benefits were observed.

## Data Availability

The raw data supporting the conclusions of this article will be made available by the authors, without undue reservation.
